# Supplementary material for: The Effect of a Structured Individualized Educational Intervention on Breastfeeding Rates in Greek Women
Source: Int J Environ Res Public Health. 2021 Oct 28;18(21):11359. doi: 10.3390/ijerph182111359 (PMC8582789; doi:10.3390/ijerph182111359)
Supplement: Supplementary file 1 [file ijerph-18-11359-s001.zip › ijerph-1386268 supplementary.pdf]

Table S1: The phases and the issues of the structured midwifery intervention in detail.

MIDWIFERY INTERVENTION	TYPE	ISSUES
1. Personalized prenatal structured midwifery education and support to low-risk pregnant and women with endocrine disorder (one session for 2 hours after 34w of gestation)	Structured midwifery education-support	a. Benefits of breastfeeding, anatomy and physiology of milk production<br>b. Breastfeeding positions, proper breastfeeding latch, importance of breastfeeding in the 1st hour after birth, breastfeeding on-demand, importance of the skin-to-skin contact and the value of rooming in instructions for women diet, management and treatment, difficulties of breastfeeding to improve maternal confidence and self-efficacy on breastfeeding
2. Personalized postnatal structured midwifery education and support to low-risk pregnant and women with endocrine disorder (one visit on the 2nd-3rd day after childbirth during their stay in the postnatal ward)	Structured midwifery education and support	a. Position and attitude of mother holding baby, method of supporting breast infant's latching on to breast when suckling, sings of effective suckling, the resolution of breastfeeding problems and direct support, the recognition of signs of nutritional adequacy of the new-born
3. Telephone communication from the midwife in charge who carried out the intervention at specified intervals (3 weeks, 4 months, 6 months, first and second year after birth)	Midwifery Telephone support	a. To empower women, support them for any difficulties of breast-feeding and provide information
